# Canine Cancer Diagnostics by X-ray Diffraction of Claws

**DOI:** 10.3390/cancers16132422

**Published:** 2024-06-30

**Authors:** Alexander Alekseev, Delvin Yuk, Alexander Lazarev, Daizie Labelle, Lev Mourokh, Pavel Lazarev

**Affiliations:** 1 Matur UK Ltd., 5 New Street Square, London EC4A 3TW, UK; aalexeev@matur.co.uk (A.A.); plazarev@matur.co.uk (P.L.); 2 Arion Diagnostics, Inc., 911 Mustang Ct, Petaluma, CA 94954, USA; delvin@ariondiagnostics.com (D.Y.); alexander@ariondiagnostics.com (A.L.); daizie@ariondiagnostics.com (D.L.); 3 Physics Department, Queens College of the City University of New York, 65-30 Kissena Blvd, Flushing, NY 11367, USA

**Keywords:** X-ray diffraction, early cancer diagnostics, structural biomarkers, canine cancer, ROC curve, keratin structure

## Abstract

**Simple Summary:**

Canine cancer is a leading cause of dog mortality. In this study, we examine the hypothesis that the structure of keratin changes when cancer develops in the patient. We use X-ray diffraction of dog claws to detect these changes, finding that the modifications of the intermolecular distances are the most significant. Machine learning algorithms are utilized for cancer/non-cancer diagnostics, achieving a balanced accuracy of 85% for the blind group. Our research suggests that the changes in keratin structure can be tracked by X-ray diffraction, offering a potential tool for non-invasive cancer diagnostics. This could have significant implications for the early detection and treatment of canine cancer, potentially saving many lives. Moreover, this approach can be extended to human cancer detection.

**Abstract:**

We report the results of X-ray diffraction (XRD) measurements of the dogs’ claws and show the feasibility of using this approach for early, non-invasive cancer detection. The obtained two-dimensional XRD patterns can be described by Fourier coefficients, which were calculated for the radial and circular (angular) directions. We analyzed these coefficients using the supervised learning algorithm, which implies optimization of the random forest classifier by using samples from the training group and following the calculation of mean cancer probability per patient for the blind dataset. The proposed algorithm achieved a balanced accuracy of 85% and ROC-AUC of 0.91 for a blind group of 68 dogs. The transition from samples to patients additionally improved the ROC-AUC by 10%. The best specificity and sensitivity values for 68 patients were 97.4% and 72.4%, respectively. We also found that the structural parameter (biomarker) most important for the diagnostics is the intermolecular distance.

## 1. Introduction

It is well known that canine cancer is the most common cause of death in dogs [1,2]. The development of methods for early cancer diagnostics helps to improve the results of cancer treatment and prolong patient life. Different techniques for canine cancer detection are available on the market, including traditional methods, such as physical examination during regular wellness visits, and the newest methods, such as genetic and molecular testing [3]. The similarity of the nature of canine and human cancers is currently used for the development of comparative and translational oncology [1,4], and the results of canine cancer studies can also be adapted to human cancer treatment.

The constantly growing market for cancer diagnostics [5] requires more efficient and reliable techniques. Standard imaging methods [6,7,8,9] include X-ray radiography, computed tomography (CT), magnetic resonance imaging (MRI), and ultrasonography. Recent developments have also introduced optical microscopy and spectroscopy (including both fluorescence and Raman spectroscopy, as well as other optical techniques) [10,11,12], electron microscopy [13], and nanotechnology-based approaches [14]. More advanced methods, such as molecular genetic testing and positron emission computed tomography, have been developed, but they still require further improvements for better accuracy and earlier diagnosis.

While the previous medical applications of X-rays were based on their absorption, we propose using X-ray diffraction (XRD) for cancer detection. For many years, XRD was used to determine the atom arrangements in crystalline solids. Later, the possibility of crystallizing biological molecules led to the development of biocrystallography, i.e., the revelation of the molecular contents of complex structures [15,16,17]. In particular, keratin’s structure was determined [18,19,20]. The XRD approach can also be applied to biological tissues without crystallization. It has been shown that it is possible to reveal the cancer-induced changes in tissues, especially in the case of breast cancer [21,22,23,24,25]. Monitoring structural changes remotely via non-invasive procedures is even more appealing for early cancer diagnostics. It has been suggested that the keratin structure of human hair is affected by cancer [26,27], but there were questions about these measurements and their interpretations [28,29]. However, XRD of remote keratin-containing locations (nails, hair, or animal whiskers) can potentially be used for high-throughput, non-invasive cancer screening in humans and animals; therefore, it is worth studying.

Recent achievements in experimental equipment, especially in the development of brighter X-ray sources and more sensitive detectors, have significantly improved the quality of diffraction data of organic materials [30]. Currently, the XRD of biological tissues is not restricted to complicated and expensive synchrotron facilities, as tabletop devices can also be utilized for fast and reliable measurements. The obtained arrays of XRD data require automated processing for further classification and diagnosis. For this purpose, artificial intelligence and machine learning (ML) algorithms have been widely applied during the last decade [31,32,33]. These novel computational algorithms significantly improve efficiency and accelerate cancer diagnostics.

In this study, we performed canine cancer detection using XRD measurements of dog claws, followed by ML modeling. The dogs under investigation were preliminarily studied through biopsy, and the obtained diagnosis was used as the target value for model development. The primary method for data processing and feature extraction used in this work was based on the Fourier series decomposition of averaged profiles of the XRD data. The calculated Fourier coefficients were utilized as features for supervised learning by the random forest classifier model. The developed algorithm implies calculating the average cancer probability per patient using several samples independently prepared and measured for each dog. The obtained results were studied and compared with the current state of the art in canine cancer diagnostics.

## 2. Materials and Methods

### 2.1. Sample Preparation and XRD Measurements

For our studies, we collected samples from 265 patients (dogs), either client-owned animals or belonging to a laboratory colony of beagles. A total of 104 patients were diagnosed with cancer, while the other 161 were not. A wide variety of breeds were represented: 31 in the healthy group and 47 in the cancer group, including several mixed-breed dogs. The dogs’ ages ranged from 0.5 to 17 years. Most of the patients had four samples, which were prepared and measured independently. The final dataset had 945 samples. None of the laboratory technicians had any information on the specifics of the samples.

**Figure 1:**
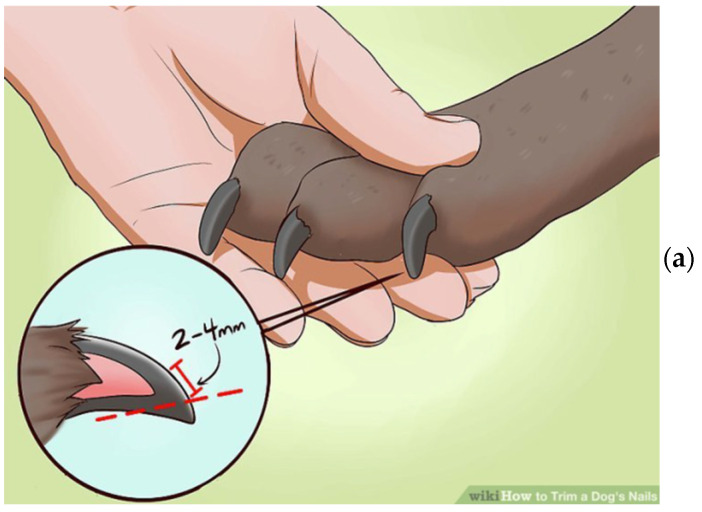

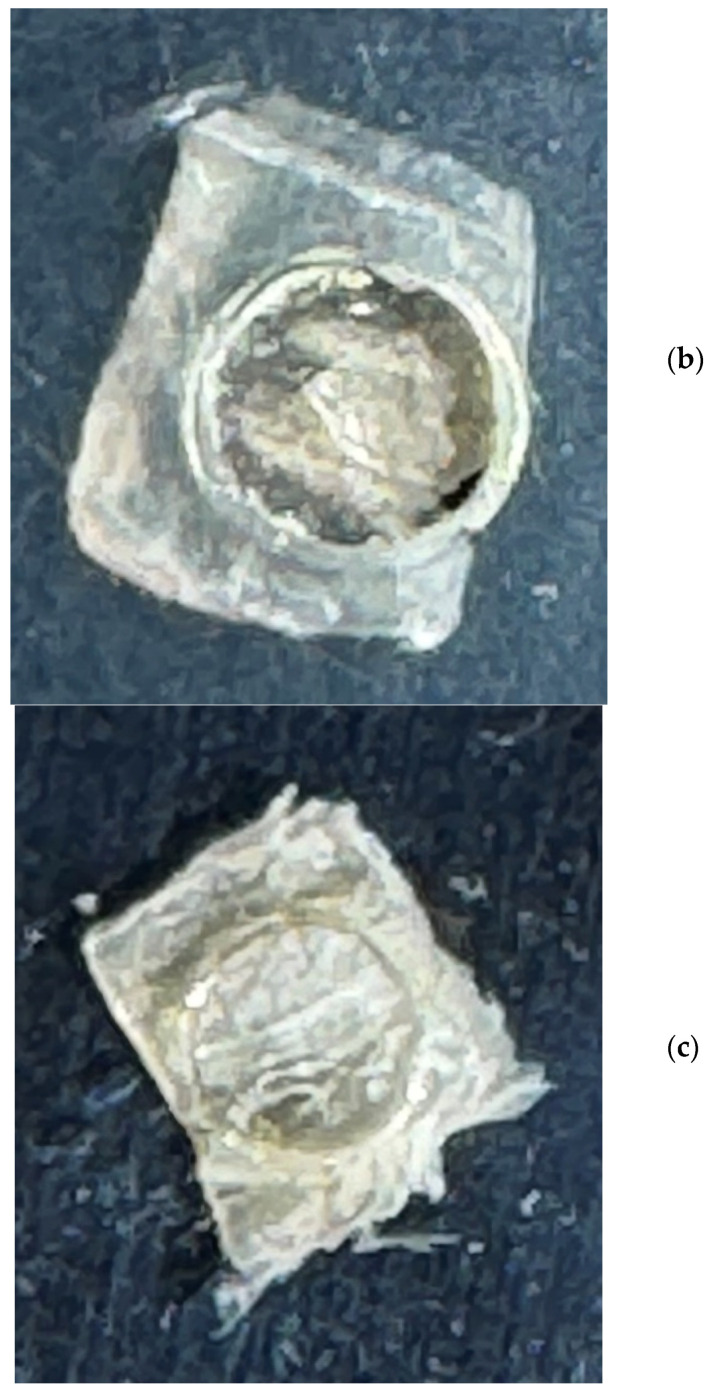
(**a**) Schematics of the nail preparation, (**b**) the nail of a cancerous dog, and (**c**) the nail of a healthy dog.

The claw specimens were previously cut to a set size and thickness using a sharp instrument. We found dog nails to include at least three visually identifiable types of tissue: (1) a hard outer shell, (2) a soft inner shell, and (3) a wax-like core, which appears to be the termination of the wick. The proper sample is a thin slice of the hard outer shell tissue, cut or shaved from the nail’s surface. To produce such samples, we used a surgical knife to cut a very thin surface layer with an area of about 3 by 3 mm (see Figure 1a). Such shavings can range roughly from 100 to 200 microns in thickness. There was no visible difference between the samples obtained from the dogs with and without cancer, as shown in Figure 1b,c. A shaving of the nail’s outer hard shell was then placed into the specially designed holder. The sample holder is intended to hold the nail shaving to be irradiated with the X-ray beam, but no part of the sample holder will interact with the beam.

The custom-developed compact X-ray diffractometer consists of three main elements: (1) the X-ray beam delivery system, (2) the sample holder receptacle, and (3) the X-ray detector. The beam delivery system contains the Xenocs X-ray source, a Genix 3D Cu with Fox 12–53 Cu Mirror, with a wavelength λ = 0.1540562 nm or energy of 8.04 keV, as well as focusing optics, also produced by Xenocs. This system delivers an X-ray beam measuring roughly 3 × 3 mm at the outlet of the focusing optics module. The output X-ray beam is focused, which means that it converges at the focal plane approximately 45 cm from the focusing optics module outlet. The focal spot size at the focal plane is specified to be 250 microns, as measured by the full width at half-maximum (FWHM).

The sample was fixed at a distance from the detector so that diffracted radiation with the transfer momentum from 2 to 13 nm^−1^ would be registered. The sample holder was placed into the receptacle on the device for measurement. The receptacle includes a circular aperture beam collimator mounted upstream (facing the X-ray source) from the sample holder. In this case, the beam is well collimated before reaching the sample. The sample holder apertures are larger than the beam, which ensures that the beam irradiates the sample without interacting with the edges of the sample holder.

The two-dimensional detector is an Advacam MiniPix SN1442 Si 500 µm with a 256-by-256-pixel array and a 55-by-55-micron pixel size. Pixels can be set to be sensitive to photons with a minimum threshold, which was 7 keV for this study. The exposure time for all samples was 2 min. The samples were measured batch by batch; all results were obtained under the same experimental conditions. The experimental data were stored as 256-by-256 matrices of integers representing the photon counts. Each batch was complemented by at least one calibration file with silver behenate (AgBH) XRD patterns.

### 2.2. Data Preprocessing

**Figure 2:**
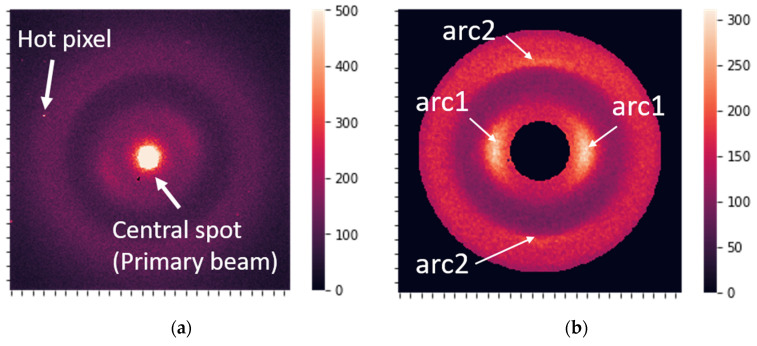
(**a**) Raw X-ray diffraction image. (**b**) The most prominent features, which correspond to the 0.51 nm periodicity associated with the molecular package (arc1) and to the 0.98 nm periodicity related to the orientation of the structural components (arc2).

The raw XRD data were 2D images (256 × 256 pixels), as shown in Figure 2a. The raw data contained hot pixels, dead pixels, a highly intensive central spot produced by the primary beam, and a background signal caused by amorphous scattering. Some additional unwanted diffraction features can appear due to the influence of the experimental setup, but not due to the sample structure. All raw images consisted of randomly oriented patterns.

Before further analysis, all of the data were preprocessed in the same way. The essential list of preprocessing steps is as follows:

1. Calibration of raw data using silver behenate (AgBH): The sample-to-detector distance may vary from batch to batch and, thus, generally requires calibration to unify scale. The image was rescaled during calibration to adjust the q-range to the same predefined standard value. The data used in the current study had a calibration spread within 3%, and calibration of these particular data was not performed.
2. Centering, cropping the central spot, and rotation of the images (CR preprocessing). The data were also cropped to a circular shape to make them symmetric.
3. Hot-spot and hot-pixel removal: Detecting hot pixels and substituting them with the average intensity value over the circle with the corresponding radius.
4. Standardizing the diffracted beam’s total intensity, i.e., the integral signal of CR-preprocessed images. The total intensity of the preprocessed images was adjusted to 5 mln counts. Typically, integral intensity is in the range of 2–10 mln counts for unnormalized images.

After preprocessing, the data were ready for analysis and contained two distinct, prominent features (Figure 2b), corresponding to periods of 0.51 and 0.98 nm in the keratin structure [20].

### 2.3. Extraction of Features

**Figure 3:**
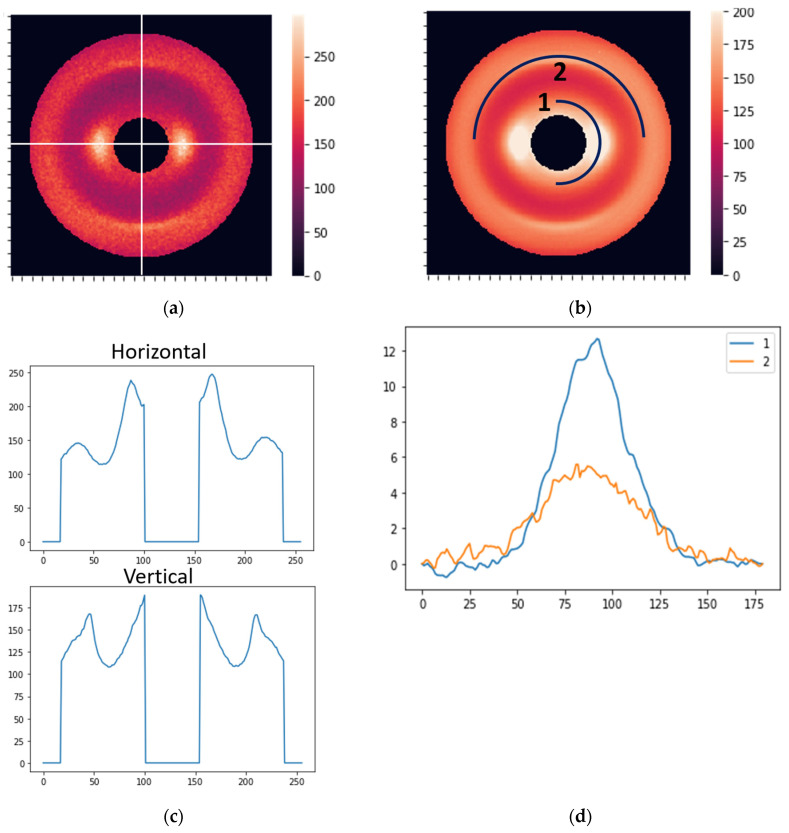
(**a**) Horizontal and vertical lines for the extraction of radial features. (**b**) Two curves for the extraction of circular features. (**c**) The distribution of the photon counts (brightness) along the horizontal and vertical cross-sections. (**d**) The intensity profiles (before normalization) corresponding to curves 1 and 2 in Figure 2b.

Our main approach to data processing was to apply the Fourier series to describe XRD images and use Fourier coefficients as features for supervised learning. Fourier coefficients accurately describe curves with arbitrary shapes. The shape of XRD profiles is usually described by well-known standard distributions like Gaussian, Lorentzian, or Voigt functions [31,32,33]. However, the XRD pattern may have complicated shapes from different sample structures. It has been shown that using the Fourier series allows for a precise approximation of the XRD profile with arbitrary shape and helps to describe image details [34,35,36,37]. In the present work, we utilize the Fourier series to describe profiles extracted from XRD images. Two types of profiles were described by the Fourier coefficients: radial and circular. The radial features were Fourier coefficients of the average curves obtained in the radial direction (direction from the center of symmetry) (Figure 3a). The circular features were extracted from curves obtained along semicircles with radii corresponding to 0.51 and 0.98 nm periods, as shown in Figure 3b. The radial features reflect distances in the molecular package, while circular features mainly indicate the orientation of structural components.

The standard N-order Fourier series expansion is given by

(1) $$S_{N}\left(x\right)=\frac{a_{0}}{2}+\sum_{n=1}^{N}\left(a_{n}\cos\frac{2\pi }{L}nx+b_{n}\sin\frac{2\pi }{L}nx\right)$$

where *L* is the *x*-range for the XRD profile, with *x* being either a radial or circular coordinate. The coefficients *a_n_* and *b_n_* can be determined and used as features in the subsequent modeling. For this study, we took *N* = 10 and used two radial directions (horizontal and vertical; see Figure 3a) and two circular curves (see Figure 3b). The coordinate dependencies of the scattered light intensity are shown in Figure 3c,d for the radial and circular directions, respectively. Accordingly, we obtained forty radial and forty circular features (ten *a_n_* and ten *b_n_* coefficients for each direction/curve).

### 2.4. Data Analysis

The final dataset had 945 samples for 265 patients (dogs), of which 161 patients did not have cancer. The cancer type was not identified during this study; thus, the binary classification “cancer”/”no cancer” was performed. A total of 68 patients (39 without cancer, 29 with cancer) were randomly selected for testing (blind dataset), while the remaining 197 patients were used for training/validation. The train/test division for the samples was 711/234. The percentage splits for patients and samples were slightly different because of the different numbers of samples per patient. The initial optimization was performed for all samples.

We used sensitivity, specificity, and balanced accuracy as performance metrics. Sensitivity is the proportion of the cancer samples that were correctly identified. Similarly, specificity measures the proportion of non-cancer samples that were correctly identified. Balanced accuracy = (specificity + sensitivity)/2. We also used the receiver operating characteristic (ROC) curve. The area under the ROC curve (ROC-AUC) is an important metric; the closer it is to one, the better. The main task for modeling was to achieve the following condition: the maximal possible balanced accuracy at a specificity greater than 90%. We also included the cancer probability threshold as a parameter of our model. This gives the value of the probability that separates cancer and non-cancer.

A model of a random forest classifier was optimized on the training dataset by using a grid of parameters and 10-fold cross-validation. The scoring of models by ROC-AUC was employed. Then, the model with the best average score was used to classify the samples from the blind (testing) dataset. The best obtained model estimated the probabilities of cancer for samples from the test group, and then the average probability per patient was calculated. Thus, the final model was obtained by transitioning from samples to patients and calculating the mean cancer probability for the patients.

The transition from sample to patient diagnostics Is not a trivial task. Most patients had a small number of wrongly classified samples, with three cancer patients having all four samples wrongly classified. Thus, these were false negatives in our model, at a threshold of 0.48. “No cancer” patients were better classified by our model, with eleven having no wrongly classified samples. These results indicate that not all claw keratin was changed during cancer development. We tried different algorithms for sample-to-patient transfer:

1. Using mean Fourier coefficients per patient. In this case, we reduced data for training and, as a result, lost information. The results obtained for mean coefficients were not stable.
2. At first, samples were classified by a supervised model, i.e., diagnosis of “cancer”/“no cancer” was predicted, and then the transfer from samples to patients was performed by using the rule “if N samples per patient are classified as cancerous, then the patient has cancer”. N can vary between 1 and 4 for patients with 4 samples. The best results were obtained for models with N = 2 or 3. The disadvantage of this method is that the same number of samples per patient is required, which was not the case due to the rejection of some images after quality control.
3. Transfer from samples to patients by averaging predicted cancer probabilities. This means that for each sample from the testing group, the cancer probability was calculated using a supervised model (random forest classifier) optimized by training samples. Then, the final classification was performed based on the probabilities averaged for each patient. A different number of samples per patient is not a problem for this model.
  Method 3 was the most successful and provided the most reliable metrics.

## 3. Results

In our modeling, we separately examined three groups of features using the same algorithm: (i) 40 radial features, (ii) 40 circular features, and (iii) both radial and circular features. The best results for classifying the randomly selected blind group of dogs are shown in Table 1. 

One can see that using only radial features produces the best results. Circular features provide reasonable metrics, showing some connection with cancer diagnosis, but this is much less efficient than a model with only radial features. The model based on radial and circular features together demonstrated metrics between the results of the models with either radial or circular features separately. The results show that the shape of the arcs in Figure 2b is much less critical for classification than the distances in the radial direction. The reduced efficiency of the model obtained with 80 features can be explained by the increased noise in circular features, which is also the result of a small number of pixels used for extracted profiles compared to averaged radial curves. In general, it can be concluded that the distances in the molecular structure of keratin are more sensitive to cancer than the orientation of structural units.

**Figure 4:**
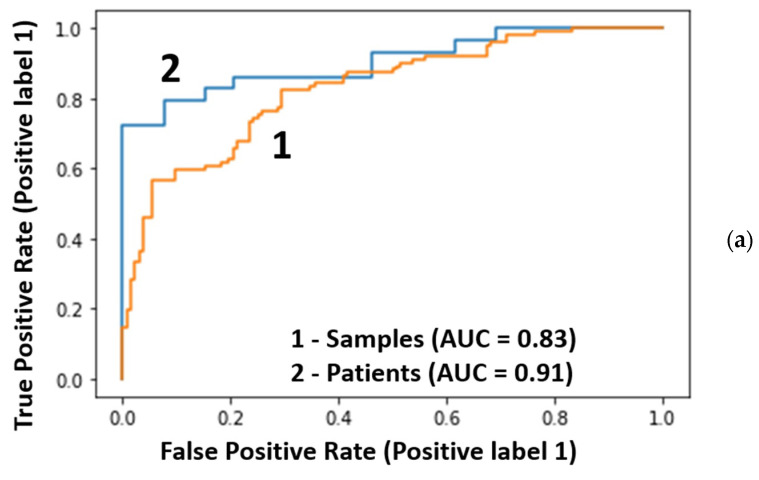

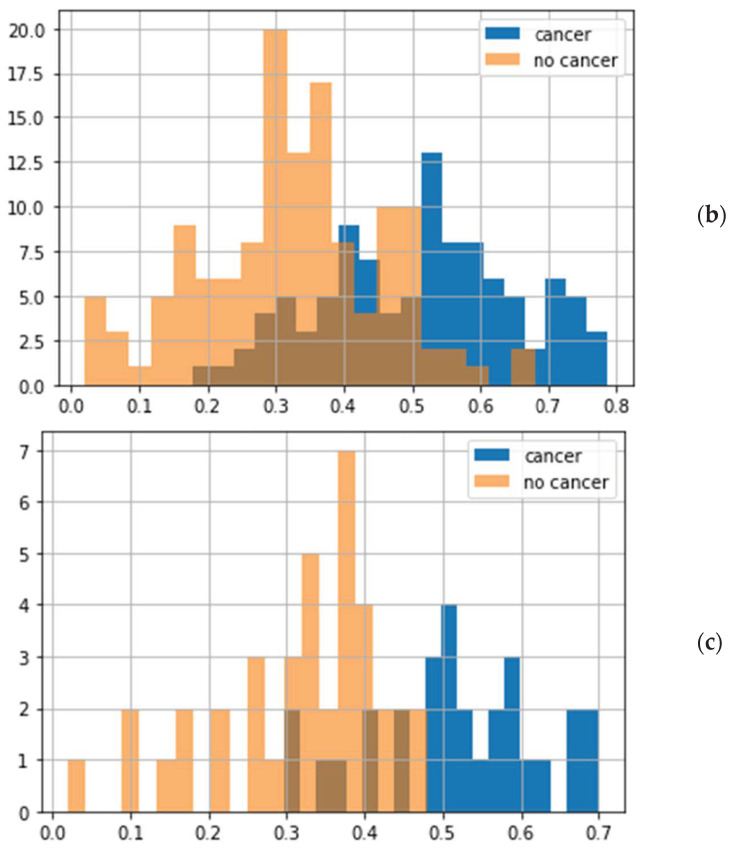
Classification of the testing data: (**a**) the ROC curves for 234 samples (orange) and 68 patients (blue), (**b**) the distribution of cancer probabilities for samples, and (**c**) the distribution of mean probabilities for patients.

The results of classifications of the test samples by the best model with 40 radial features are shown in Figure 4a,b and Table 1. For the blind set, ROC-AUC = 0.83, and the histogram with the distribution of probabilities for the samples has significantly overlapping groups of cancer/no cancer samples (Figure 4b). Transition to the descriptions in terms of patients is shown in Figure 4c.

Significant improvement of the metrics was observed after transfer from samples to patients: the ROC-AUC increased from 0.83 to 0.91 (Figure 4a). This increase in ROC-AUC value was also observed for the other two models (Table 1).

**Figure 5:**
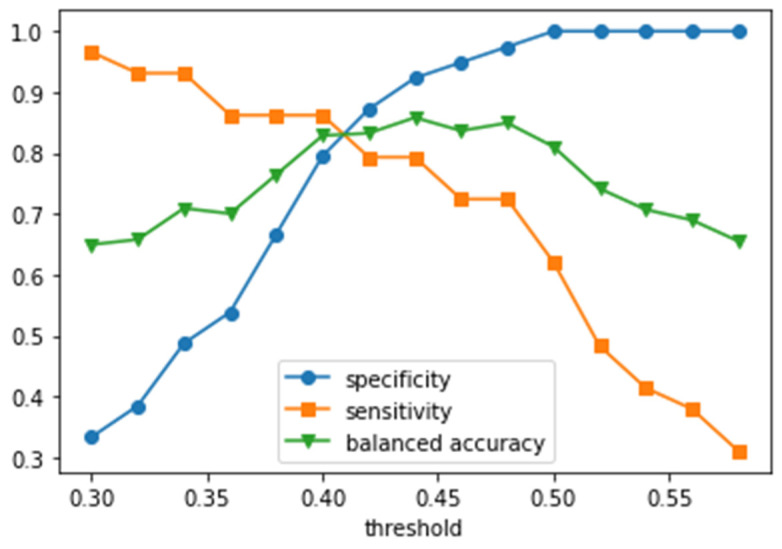
Specificity, sensitivity, and balanced accuracy as functions of the mean probability threshold per patient.

The dependencies of specificity and sensitivity on the probability threshold for cancer diagnosis by the best obtained model are shown in Figure 5. The maximal balanced accuracy value of 84.9% at the specificity of 97.4% was achieved at the threshold of 0.48 (the balanced accuracy at the threshold of 0.44 was 85.8, but the specificity was lower than one at the threshold of 0.44). In a wide range of thresholds (0.4–0.5), the balanced accuracy exceeded 80%. The exact threshold value for clinical practice should be chosen depending on conditions and tasks: thresholds in the range of 0.42–0.48 provide maximal balanced accuracy; at higher thresholds, specificity increases while balanced accuracy decreases (Figure 5).

## 4. Discussion

**Figure 6:**
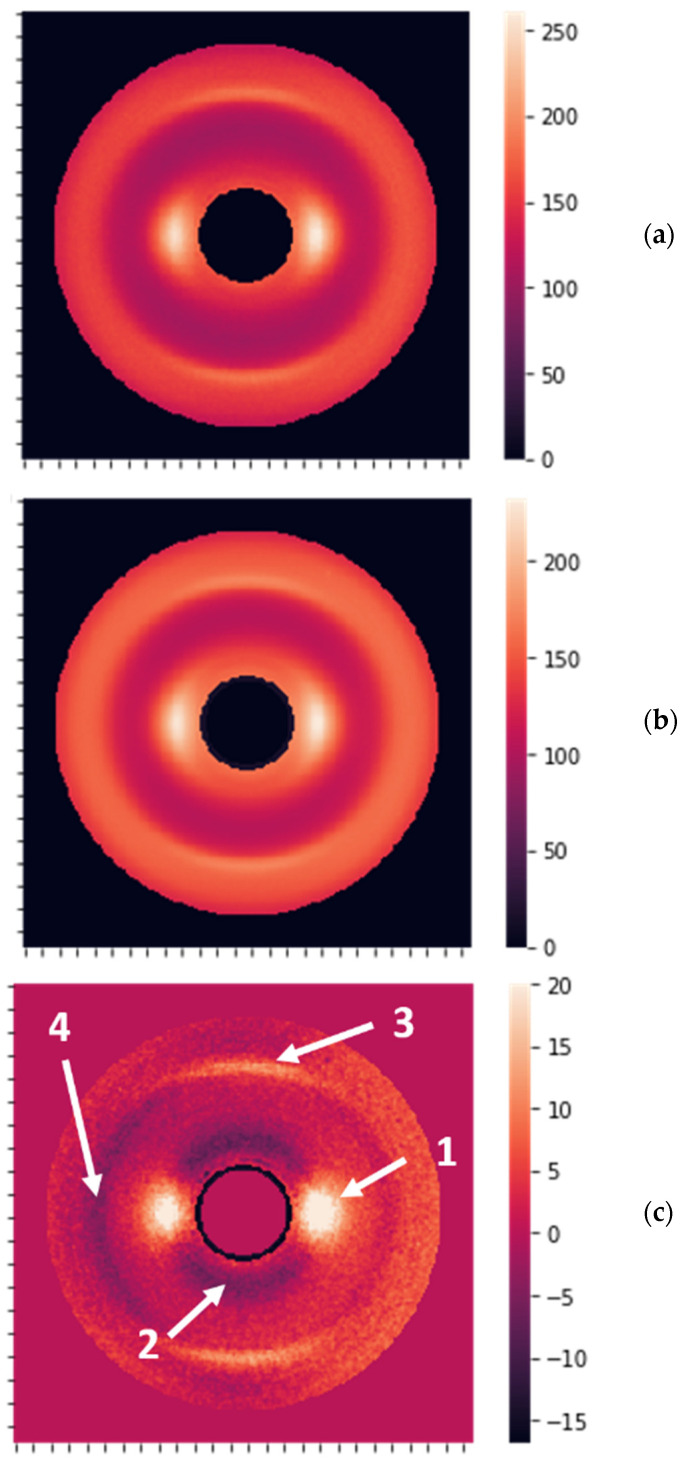
(**a**) The image obtained by averaging over 53 “no cancer” XRD patterns. (**b**) The image obtained by averaging over 339 “cancer” XRD patterns. (**c**) The difference between averaged “no cancer” and “cancer” images, with the most prominent features shown by the arrows 1, 2, 3, and 4.

The obtained results can be better understood by connecting the metrics to the properties of the images and determining the most essential features. The averaged “no cancer” and “cancer” images are shown in Figure 6a,b. Figure 6c reveals the areas affecting the diagnosis.

The most significant differences were observed for the peaks at 0.98 nm (arrow 1 in Figure 6c) and the ring at the same distance from the center of symmetry (arrow 2). The opposite sign for these differences implies that the peaks are higher for non-cancer samples, while the rings are more pronounced for the cancer samples. Additionally, there are fewer visible differences at 0.51 nm (arrows 3 and 4). The peaks at 0.98 nm correspond to the packing of long keratin molecules. In the healthy state, hydrogen bonds are directed outside the molecules, dictating the packing distance. When the structure is compromised by the cancerous state, these hydrogen bonds can drop the water and cross-connect the molecules, breaking the packing. This process leads to the suppression of the peaks and can serve as a structural biomarker, i.e., it can be used for cancer detection.

The obtained results offer a fast and convenient early canine cancer detection method by using XRD measurements of claws or hairs and other keratin-containing tissues. Recently developed and quickly disseminating liquid biopsy methods achieved specificity/sensitivity = 98.5%/54.7% [38,39]. Here, we demonstrate the best result of 97.4%/72.4% for the randomly selected blind testing group of patients. This result is better in terms of balanced accuracy: 84.9% here vs. 76.6% by liquid biopsy. Thus, our introduced method, which includes XRD measurements of keratin structure in claws, calculation of Fourier coefficients, supervised learning by using samples, and calculation of mean cancer probability per patient, can detect cancer with promising efficiency; consequently, keratin’s structure can be discussed as a promising cancer biomarker. Further development of this method should include unification and optimization of the measurement protocol, the use of the same and sufficient number of samples per patient, an increased number of patients for modeling, more precise calibration of XRD results, etc. The transition from 1D to 2D Fourier series can be considered as the next step in algorithm development. Experimental improvements, e.g., a detector with higher resolution and possibilities for measurements in a controlled atmosphere, will also be beneficial.

## 5. Conclusions

We reported the feasibility of the X-ray diffraction measurements of dogs’ claws for cancer detection. The data were analyzed to achieve the maximum possible balanced accuracy at a specificity greater than 90%. Our best result provided balanced accuracy for 68 patients, achieving 84.9%, with the best specificity and sensitivity values being 97.4% and 72.4%, respectively. This exceeds the current liquid biopsy results. For our binary classification, we used the Fourier coefficients of four profiles of XRD data: two in radial directions and two in circular directions. The introduced transition method from samples to patients by averaging the mean cancer probability per patient demonstrated a ~10% increase in ROC-AUC value. We found that the distances in the molecular structure are more influenced by cancer than by the orientation of structural units. However, more studies are necessary before our approach can be widely used in veterinary practice. In particular, more samples and patients are required for a more reliable and stable analytic model, and a better signal-to-noise ratio is desirable to improve the feature extraction.

## Figures and Tables

**Table 1:** The best results for the blind group with 39 no-cancer patients and 29 cancer patients.

	40 Radial Features	40 Circular Features	40 Radial and 40 Circular Features
Threshold	0.48	0.46	0.46
Specificity, %	97.4	92.3	94.9
Sensitivity, %	72.4	44.8	58.6
Balanced accuracy, %	84.9	68.6	76.7
ROC-AUC (samples/patients)	0.83/0.91	0.69/0.77	0.80/0.87

## Data Availability

The authors will make the raw data and codes available upon request.
